# Pulse rate variability and health-related quality of life assessment with the Short Form-8 Japanese version in the general Japanese population

**DOI:** 10.1038/s41598-024-54748-9

**Published:** 2024-02-20

**Authors:** Isao Saito, Koutatsu Maruyama, Kanako Yamauchi, Yayoi Funakoshi, Tadahiro Kato, Ryoichi Kawamura, Yasunori Takata, Haruhiko Osawa

**Affiliations:** 1 Department of Public Health and Epidemiology, Faculty of Medicine, Oita University, 1-1 Idaigaoka, Hasama-machi, Yufu, Oita 879-5593 Japan; 2 Department of Bioscience, Graduate School of Agriculture, Ehime University, Matsuyama, Ehime Japan; 3 Faculty of Education, Fukuyama City University, Fukuyama, Hiroshima Japan; 4 Division of Life Span Development and Clinical Psychology, Graduate School of Education, Ehime University, Matsuyama, Ehime Japan; 5 Department of Diabetes and Molecular Genetics, Ehime University Graduate School of Medicine, Toon, Ehime Japan

**Keywords:** Biomarkers, Epidemiology, Outcomes research

## Abstract

We aimed to investigate the association between pulse rate variability (PRV) and health-related quality of life (HRQOL) in the general population. A cross-sectional study was conducted with 5908 Japanese men and women aged 30–79 years. PRV was assessed at rest using 5-min recordings of pulse waves with a photoplethysmographic signal from a fingertip sensor, and the time and frequency domains of PRV were determined. HRQOL was assessed with the Short Form-8 (SF-8) Japanese version, and poor HRQOL was defined as an SF-8 sub-scale score < 50. A test for nonlinear trends was performed with the generalized additive model with a smoothing spline adjusted for confounders. The lowest multivariable-adjusted odds ratios for poor physical component score were found in those who had second or third quartile levels of standard deviation of normal-to-normal intervals (SDNN) and root mean square of successive difference (RMSSD), and high-frequency (HF) power and trended slightly upward in the higher levels. PRV-derived parameters were nonlinearly associated with poor physical component scores. In conclusion, reduced PRV-derived SDNN, RMSSD and HF power were associated with poor HRQOL in the domain of physical function. Higher levels of these parameters did not necessarily translate into better HRQOL.

## Introduction

Health-related quality of life (HRQOL) is measured as an outcome for chronic disease and medical evaluations in clinical settings and has been measured in the general population^1,2^. HRQOL scores consist of physical and mental components and are decreased in individuals with low physical activity^3^, insomnia^4^, diabetes^5^, and hypertension^6^. Furthermore, poor self-rated health, which is one of the domains of HRQOL^7^, strongly predicts mortality and major outcomes^8–10^.

Heart rate variability (HRV) parameters are indicators of autonomic nervous system function^11^. These indicators represent sympathetic and parasympathetic nervous system activity and balance, and cohort studies have reported that autonomic nervous system dysfunction increases the risk of mortality^12^ and is an independent risk factor for incident diabetes^13,14^ and cardiovascular disease^15,16^. Alternatively, a systematic review found that increases in levels of endocrine hormones, such as cortisol, and decreases in HRV were biomarkers of psychological stress, with various studies showing an association between workplace stressors and HRV^17^.

Pulse rate variability (PRV) has been used as a potential surrogate marker of HRV and is measured with photoplethysmography. A review of studies that compared photoplethysmography with electrocardiography found that PRV accurately reflects HRV conditions for healthy subjects when measured at rest^18^. However, some studies indicated that PRV did not agree with the results from HRV under certain conditions, such as body posture and exercise, suggesting that PRV may be a useful biomarker distinct from HRV^19^. In line with this, a recent review has suggested that it would be difficult to assume that PRV is a validated surrogate for HRV, rather that HRV may reflect another aspect of cardiovascular dynamics^20^.

Poor HRQOL is usually explained by mental and physical conditions in individuals suffering from chronic diseases or stressors as a whole^2^. Consequently, poor HRQOL is associated with an increased risk of mortality and other major outcomes^10^. However, the biological mechanisms by which poor HRQOL influences these outcomes have not been established^21^.

Some studies have demonstrated an association between HRV and HRQOL in patients with functional somatic syndrome^22^, intellectual disabilities^23^, and elderly individuals with cognitive impairment^24^. Another study reported an association between HRV and HRQOL in 329 healthy volunteers aged 20–54 years and found that poor HRQOL in the physical domain was associated with a reduction in HRV^25^. Nonetheless, evidence from the general population is limited.

Although there is some debate as to whether the PRV-derived parameters reflect autonomic function or another circulatory dynamics, previous studies that applied PRV in several settings suggested that PRV-derived parameters represent some aspects of health status^20^. Therefore, the purpose of this study was to examine the relationship between these indices and HRQOL in the general population.

## Methods

### Study subjects

We enrolled 6013 men and women, 30–79 years of age, who lived in Ozu and Toon Cities in Ehime prefecture, Japan, from 2009 to 2012 and 2014–2018 (Toon City only). Both cities are in rural areas of Shikoku Island Japan. We excluded subjects who did not have a PRV examination (n = 33) and who had atrial fibrillation on an electrocardiogram (ECG) (n = 37). After the exclusion of those who did not respond to the HRQOL questionnaire (n = 36), 5908 individuals remained and were included in the analysis.

This study was conducted in accordance with the Declaration of Helsinki. Written informed consent was obtained from all participants. The study protocol was approved by the Human Ethics Review Committee of the Faculty of Medicine, Oita University (approval number, 2114).

### Measurements

Blood pressure was measured twice with an automatic sphygmomanometer (BP-103iII; OMRON Colin Co., Tokyo, Japan) with the subject in the sitting position after a rest of at least 5 min. We used the mean of the two measurements for the analysis. The use of antihypertensive drugs was ascertained by questionnaire. Diabetes was defined as hemoglobin A1c (HbA1c) ≥ 6.5% or current use of anti-diabetes agents. HbA1c was measured with high-performance liquid chromatography (Ozu City) and the immunoagglutination inhibition method (Toon City). Body mass index (BMI) was calculated as weight (kg) divided by height (m) squared.

HRQOL was assessed with the Short Form-8 (SF-8) Japanese version questionnaire^1^, which consists of eight subscales—general health (GH), physical functioning (PF), role physical (RP), bodily pain (BP), vitality (VT), social functioning (SF), mental health (MH), and role emotional (RE)—and two summary scores—the physical component score (PCS), which consists of GH, PF, RP, and BP and the mental component score (MCS), which consists of VT, SF, MH, and RE. All subscales and summary scores were standardized in the general Japanese population (mean = 50 and standard deviation = 10). In the present study, poor HRQOL was defined as a score of less than 50 for each subscale and summary score.

A self-administered questionnaire was used to assess medical history (heart disease, stroke, and kidney disease), smoking habits (≥ 20 cigarettes/day, 1–19 cigarettes/day, past smoker, and never smoker), regular alcohol drinking, exercise habits, and sleep duration. Current smokers were defined as individuals who had smoked 100 cigarettes in their lifetime and who currently smoked cigarettes. Exercise was defined as doing continuous sports or physical exercise ≥ 2 times/week during the year. Unhealthy sleep duration was defined as < 6-h or ≥ 9-h of sleep per day^26^.

### Assessment of autonomic function

Analysis of PRV was performed with the TAS9 device (YKC Co. Ltd, Japan) and its software to assess cardiac autonomic control. The pulse rate was recorded for 5 min with a fingertip pulse wave sensor using a photoplethysmographic signal. The sensor was attached to the index finger, with the subject in the sitting position after a rest of at least 5 min, with the software filtering out arrhythmias and artifacts. Filtering was based on the Butterworth bandpass filter method to detect the normal-to-normal intervals accurately^27^. The five-minute PRV measurement procedure, including PRV indices and standardization, was based on the recommendation of the European Society of Cardiology and the North American Society of Pacing and Electrophysiology^28^. The Task Force recommends appropriate HRV parameters in the time and frequency domains and states that five-minute recordings with a stationary system are preferable. The sampling frequency for all recordings was 1000 Hz. PRV obtained from the fingertip pulse wave sensor is comparable with that of an ECG signal^29^.

All PRV assessments were done between 10:00 am and 12:00 pm to control for daily variation. We maintained a comfortable room temperature with air conditioners. Participants were required to have at least a five-minute rest before the measurement. The TAS9 software-provided resting heart rate (RHR) was expressed as beats per minute (bpm), and the following time-domain measures of PRV were then determined: standard deviation of normal-to-normal intervals (SDNN), root mean square of successive difference (RMSSD), and percentage differences between normal NN intervals > 50 ms (pNN50). PRV in the frequency domain was calculated using the fast Fourier Transform with a sample rate of 3 Hz. The area under the curve fitted by the power spectral density was measured for the frequency bands of low-frequency (LF) power (0.04–0.15 Hz) and high-frequency (HF) power (0.15–0.40 Hz).

In general, SDNN reflects the total modulation of autonomic function, while RMSSD and pNN50 reflect vagal control of the heart. LF power reflects both sympathetic and parasympathetic stimulation of the heart, and HF power reflects vagal activity^28^.

Although PRV-derived parameters were determined based on five-minute recordings, we confirmed that they were significantly correlated with those provided by 24-h Holter ECG assessment^30^.

### Statistical analysis

Because of their skewed distribution, SDNN, RMSSD, LF power, and HF power were log-transformed before analysis. An analysis was also carried out to measure differences in the means of age, BMI, RHR, systolic blood pressure (SBP), and the proportions of men and women, subjects with hypertension, diabetes, smoking, regular alcohol drinking, regular exercise, unhealthy sleep duration, and medical history, grouped by PCS and MCS (poor versus good conditions). The differences between groups were tested with a t-test or chi-square test. While stratified by community, we created a sex and age-adjusted model, and a multivariable model including covariates, i.e., BMI (continuous), hypertension (Yes or No), diabetes (Yes or No), smoking (≥ 20 cigarettes, 1–19 cigarettes, past smoker, and never smoker), regular alcohol drinking (Yes or No), regular exercise (Yes or No), unhealthy sleep duration (Yes or No), and medical history (Yes or No) and to estimate odds ratios (ORs) and 95% confidence intervals (Cis) for poor HRQOL. Trend tests were performed in the two models with linear or quadratic equations for a linear or nonlinear trend test, respectively. To illustrate the nonlinear trends of lnSDNN, lnRMSSD, lnLF power, and lnHF power for poor HRQOL, the generalized additive model (GAM) with a smoothing spline [degree of freedom = 3] was adopted in the logistic regression model^31^, adjusting for covariates in the multivariable-adjusted model. Statistical significance was assumed at *P* < 0.05. All statistical analyses were performed with SAS software, version 9.4 (SAS Institute, Inc., Cary, NC, USA).

## Results

Table 1 shows the comparison of variables for cardiovascular risk factors and lifestyle between groups with poor and good PCS and MCS. The mean age was higher in the poor PCS group and lower in the poor MCS group. Individuals in the poor PCS group had higher means or percentages of BMI, SBP, diabetes, anti-hypertensive drug use, and medical history. PRV-derived parameters were significantly lower in the poor PCS group except for RMSSD. On the other hand, the poor MCS group had lower levels of BMI and SBP, and a significant difference in PRV-derived parameters between groups was not seen. The percentage of subjects with regular exercise habits was significantly lower in both the poor PCS and MCS groups compared with the good PCS and MCS groups, and that of subjects with unhealthy sleep duration was significantly higher in both the poor PCS and MCS groups compared with the good PCS and MCS groups.

**Table 1:** Population characteristics according to physical and mental component scores.

	Physical component score		*P* value	Mental component score		*P* value
	Poor	Good		Poor	Good	
Number of subjects	3237	2671		2626	3282	
Male sex, n (%)	1204 (37.2)	1094 (41.0)	0.003	924 (35.2)	1374 (41.9)	< 0.001
Age, years	62.2 ± 10.4	60.3 ± 10.7	< 0.001	59.7 ± 11.2	62.6 ± 9.9	< 0.001
Body mass index, kg/m^2^	23.3 ± 3.4	22.8 ± 3.1	< 0.001	22.9 ± 3.3	23.2 ± 3.3	< 0.001
Resting heart rate, bpm	68.9 ± 10.3	68.4 ± 9.7	0.025	69.2 ± 9.9	68.3 ± 10.1	0.001
Systolic blood pressure, mmHg	129.7 ± 19.4	128.2 ± 19.8	0.005	127.5 ± 19.7	130.2 ± 19.4	< 0.001
Hypertension, n (%)	1535 (47.4)	1039 (38.9)	< 0.001	1066 (40.6)	1508 (46.0)	< 0.001
Diabetes, n (%)	272 (8.4)	161 (6.0)	< 0.001	186 (7.1)	247 (7.5)	0.52
Anti-hypertensive drug use, n (%)	967 (29.9)	544 (20.4)	< 0.001	635 (24.2)	876 (26.7)	0.028
Smoking status, n (%)						
Never smoker	2311 (71.4)	1853 (69.4)	0.26	1894 (72.1)	2270 (69.2)	0.070
Past smoker	588 (18.2)	531 (19.9)		461 (17.6)	658 (20.1)	
1–19 cigarettes/day	126 (3.9)	117 (4.4)		103 (3.9)	140 (4.3)	
≥ 20 cigarettes/day	212 (6.6)	170 (6.4)		168 (6.4)	214 (6.5)	
Regular alcohol drinker, n (%)	1519 (46.9)	1357 (50.8)	0.003	1259 (47.9)	1617 (49.3)	0.31
Regular exercise, n (%)	1441 (44.5)	1345 (50.4)	< 0.001	1157 (44.1)	1629 (49.6)	< 0.001
Unhealthy sleep duration, n (%)	504 (15.6)	313 (11.7)	< 0.001	416 (15.8)	401 (12.2)	< 0.001
Medical histories, n (%)						
Heart disease	303 (9.4)	139 (5.2)	< 0.001	202 (7.7)	240 (7.3)	0.58
Stroke	137 (4.2)	77 (2.9)	0.006	86 (3.3)	128 (3.9)	0.20
Kidney disease	9 (0.3)	8 (0.3)	0.88	10 (0.4)	7 (0.2)	0.23
PRV parameters						
lnSDNN, ms	3.61 ± 0.52	3.65 ± 0.48	0.004	3.63 ± 0.50	3.63 ± 0.50	0.77
lnRMSSD, ms	3.38 ± 0.67	3.39 ± 0.63	0.34	3.37 ± 0.64	3.39 ± 0.65	0.161
pNN50, %	47.8 ± 18.9	50.0 ± 18.1	< 0.001	49.3 ± 18.9	48.5 ± 18.3	0.115
lnLF power, ms^2^	4.99 ± 1.33	5.12 ± 1.23	< 0.001	5.06 ± 1.27	5.05 ± 1.30	0.82
lnHF power, ms^2^	4.63 ± 1.35	4.70 ± 1.24	0.033	4.67 ± 1.28	4.65 ± 1.31	0.55

*PRV* pulse rate variability, *SDNN* standard deviation of the normal-to-normal intervals, *RMSSD* root mean square of the successive differences of NN intervals, *pNN50* percentage differences between normal NN intervals > 50 ms, *LF* low-frequency, and *HF* high-frequency.

Sex- and age-adjusted ORs and 95% CIs for poor HRQOL subscales according to quartile of PRV parameter (as referenced to the lowest quartile) and results of linear and nonlinear tests are presented (S1 and S2 Tables). ORs of RHR for subjects with the poor GH, PF, RP, and VT increased linearly, and the OR of RHR for 80 + bpm versus < 60 bpm in individuals with poor VT increased to 1.56 (95% CI 1.29–1.89). The association between SDNN and poor GH, PF, RP, VT, SF, and RE showed a significant nonlinear trend rather than a linear trend. Similarly, for RMSSD, LF power, and HF power, there were significant nonlinear trends toward the poor SF-8 subscale groups.

Tables 2 and 3 show multivariable-adjusted ORs and 95% CIs and results of linear and nonlinear trend tests after multivariable adjustment. The linear association between RHR; and poor GH, PF, RP, and VT remained significant after adjustment for covariates. Nonlinear trends in SDNN, RMSSD, and HF power were found for poor GH, PF, and RP. The nonlinear trends in SDNN, RMSSD, LF power, and HF power for poor VT weakened consistently; however, the ORs of RMSSD and HF power and the nonlinear trends remained significant.

**Table 2:** Multivariable-adjusted ORs and 95% CIs according to RHR levels and quartile of PRV parameters for poor general health, physical functioning, role physical, and bodily pain (n = 5908).

Parameter	Category	Subscale of SF-8 (Multivariable-adjusted models)							
		General health		Physical function		Role physical		Bodily pain	
		OR	95% CI	OR	95% CI	OR	95% CI	OR	95% CI
RHR	< 60 bpm	1.00		1.00		1.00		1.00	
	60–69	1.15	0.95–1.38	1.16	1.00–1.36	1.07	0.92–1.25	1.02	0.88–1.18
	70–79	1.21	0.99–1.47	1.08	0.91–1.27	1.07	0.91–1.26	0.99	0.84–1.16
	80 +	1.27	1.00–1.60	1.25	1.02–1.52	1.19	0.98–1.44	1.04	0.86–1.26
	Linear *P*	0.017		0.035		0.036		0.95	
	Nonlinear *P*	0.33		0.092		0.147		0.92	
SDNN	Q1 (lowest)	1.00		1.00		1.00		1.00	
	Q2	0.82	0.69–0.98	0.82	0.70–0.95	0.79	0.68–0.92	0.92	0.80–1.07
	Q3	0.84	0.71–1.01	0.93	0.80–1.09	0.94	0.81–1.10	0.95	0.82–1.11
	Q4 (highest)	0.86	0.72–1.03	0.84	0.72–0.98	0.78	0.67–0.90	0.96	0.83–1.12
	Linear *P*	0.90		0.57		0.076		0.94	
	Nonlinear *P*	< 0.001		< 0.001		< 0.001		0.96	
RMSSD	Q1 (lowest)	1.00		1.00		1.00		1.00	
	Q2	0.87	0.73–1.03	0.87	0.73–1.03	0.89	0.77–1.03	0.93	0.80–1.07
	Q3	0.89	0.75–1.06	0.89	0.75–1.06	0.91	0.78–1.05	1.03	0.89–1.19
	Q4 (highest)	0.82	0.69–0.98	0.82	0.69–0.98	0.91	0.79–1.06	0.96	0.83–1.11
	Linear *P*	0.39		0.92		0.30		0.61	
	Nonlinear *P*	0.004		< 0.001		< 0.001		0.72	
pNN50	Q1 (lowest)	1.00		1.00		1.00		1.00	
	Q2	0.96	0.80–1.14	0.84	0.73–0.98	0.85	0.73–0.98	1.10	0.95–1.27
	Q3	0.93	0.78–1.11	0.88	0.76–1.03	0.87	0.75–1.01	0.99	0.86–1.15
	Q4 (highest)	0.76	0.64–0.92	0.86	0.73–1.00	0.87	0.74–1.01	1.01	0.87–1.18
	Linear *P*	0.002		0.018		0.023		0.61	
	Nonlinear *P*	0.35		0.006		0.001		0.59	
LF power	Q1 (lowest)	1.00		1.00		1.00		1.00	
	Q2	0.77	0.64–0.92	0.78	0.67–0.90	0.86	0.75–1.00	0.92	0.80–1.07
	Q3	0.79	0.66–0.95	0.85	0.73–0.99	0.90	0.77–1.04	0.87	0.75–1.01
	Q4 (highest)	0.81	0.68–0.97	0.86	0.73–1.00	0.83	0.72–0.97	0.93	0.80–1.08
	Linear *P*	0.18		0.20		0.050		0.20	
	Nonlinear *P*	0.014		< 0.001		0.079		0.81	
HF power	Q1 (lowest)	1.00		1.00		1.00		1.00	
	Q2	0.78	0.65–0.93	0.79	0.68–0.91	0.80	0.69–0.93	0.87	0.75–1.01
	Q3	0.79	0.66–0.94	0.86	0.74–1.00	0.82	0.71–0.96	0.85	0.73–0.99
	Q4 (highest)	0.82	0.69–0.98	0.91	0.78–1.06	0.88	0.75–1.02	0.90	0.78–1.05
	Linear *P*	0.40		0.98		0.21		0.22	
	Nonlinear *P*	0.001		< 0.001		< 0.001		0.76	

ORs were adjusted for sex, age, BMI, hypertension, diabetes, smoking, regular alcohol drinking, regular exercise, unhealthy sleep duration, and medical histories stratified by community. Ranges: SDNN (ms), Q1: < 3.30 (log), Q2: 3.30–3.58, Q3: 3.59–3.91, Q4: 3.92+; RMSSD (ms), Q1: < 3.00 (log), Q2: 3.00–3.32, Q3: 3.33–3.73, Q4: 3.74+; pNN50 (%), Q1: < 37, Q2: 37–50, Q3: 51–62, Q4: 63+; LF (ms^2^), Q1: < 4.23 (log), Q2: 4.23–4.94, Q3: 4.95–5.80, Q4: 5.81+; and HF (ms^2^), Q1: < 3.83 (log), Q2: 3.83–4.50, Q3: 4.51–5.30, Q4: 5.31+

*OR* odds ratio, *CI* confidence interval, *RHR* resting heart rate, *PRV* pulse rate variability, *SDNN* standard deviation of the normal-to-normal intervals, *RMSSD* root mean square of the successive differences of NN intervals; pNN50, percentage differences between normal NN intervals > 50 ms; *LF* low-frequency, and *HF* high-frequency.

**Table 3:** Multivariable-adjusted ORs and 95% CIs according to RHR levels and quartile of PRV parameters for poor vitality, social functioning, mental health, and role emotional (n = 5908).

Parameter	Category	Subscale of SF-8 (Multivariable-adjusted models)							
		Vitality		Social functioning		Mental health		Role emotional	
		OR	95% CI	OR	95% CI	OR	95% CI	OR	95% CI
RHR	< 60 bpm	1.00		1.00		1.00		1.00	
	60–69	1.18	1.01–1.38	1.03	0.88–1.21	1.21	1.02–1.43	1.17	1.01–1.36
	70–79	1.32	1.12–1.56	1.11	0.94–1.32	1.24	1.04–1.48	1.28	1.09–1.51
	80+	1.56	1.28–1.90	1.09	0.89–1.34	1.23	1.00–1.52	1.12	0.92–1.36
	Linear *P*	< 0.001		0.172		0.077		0.131	
	Nonlinear *P*	0.80		0.68		0.38		0.096	
SDNN	Q1 (lowest)	1.00		1.00		1.00		1.00	
	Q2	0.83	0.72–0.96	0.89	0.76–1.04	0.92	0.79–1.08	0.90	0.78–1.04
	Q3	0.85	0.73–0.99	0.97	0.83–1.14	0.88	0.75–1.03	0.93	0.80–1.08
	Q4 (highest)	0.84	0.72–0.97	0.94	0.81–1.10	0.86	0.73–1.01	0.88	0.75–1.02
	Linear *P*	0.080		0.47		0.121		0.123	
	Nonlinear *P*	0.23		< 0.001		0.001		0.027	
RMSSD	Q1 (lowest)	1.00		1.00		1.00		1.00	
	Q2	0.82	0.71–0.95	0.92	0.79–1.07	0.83	0.71–0.97	0.89	0.76–1.02
	Q3	0.89	0.77–1.03	0.89	0.76–1.04	0.81	0.70–0.95	0.83	0.71–0.96
	Q4 (highest)	0.81	0.69–0.93	0.92	0.79–1.08	0.83	0.71–0.97	0.83	0.72–0.96
	Linear *P*	0.054		0.41		0.042		0.031	
	Nonlinear *P*	0.294		< 0.001		0.143		0.022	
pNN50	Q1 (lowest)	1.00		1.00		1.00		1.00	
	Q2	0.92	0.79–1.06	0.84	0.72–0.98	0.91	0.78–1.07	0.94	0.81–1.09
	Q3	0.86	0.74–1.00	0.88	0.76–1.03	0.86	0.73–1.01	0.87	0.75–1.01
	Q4 (highest)	0.81	0.70–0.95	0.84	0.72–0.99	0.88	0.75–1.03	0.87	0.74–1.01
	Linear *P*	< 0.001		0.033		0.065		0.053	
	Nonlinear *P*	0.26		0.033		0.51		0.63	
LF power	Q1 (lowest)	1.00		1.00		1.00		1.00	
	Q2	0.88	0.75–1.02	0.88	0.75–1.03	0.86	0.74–1.01	0.83	0.72–0.97
	Q3	0.90	0.77–1.05	0.89	0.76–1.04	0.83	0.71–0.97	0.82	0.71–0.96
	Q4 (highest)	0.88	0.75–1.03	0.81	0.69–0.95	0.85	0.72–1.00	0.84	0.72–0.98
	Linear *P*	0.043		0.038		0.029		0.041	
	Nonlinear *P*	0.011		0.149		0.42		0.36	
HF power	Q1 (lowest)	1.00		1.00		1.00		1.00	
	Q2	0.77	0.66–0.89	0.89	0.76–1.04	0.88	0.75–1.03	0.89	0.77–1.04
	Q3	0.83	0.71–0.97	0.92	0.79–1.08	0.83	0.71–0.97	0.89	0.77–1.03
	Q4 (highest)	0.80	0.69–0.93	0.94	0.80–1.10	0.92	0.79–1.08	0.90	0.78–1.05
	Linear *P*	0.027		0.38		0.144		0.160	
	Nonlinear *P*	0.011		0.062		0.38		0.35	

Table footnote is the same as Table 2.

Regarding the summary component of PCS and MCS, SDNN, RMSSD, and HF parameters were associated with poor PCS in nonlinear manners (Table 4). On the other hand, the associations were not clear for poor MCS.

**Table 4:** Multivariable-adjusted ORs and 95% CIs according to RHR levels and quartile of PRV parameters for poor physical component score and mental component score (n = 5908).

Parameter	Category	Subscale of SF-8 (Multivariable-adjusted models)			
		Poor physical component score		Poor mental component score	
		OR	95% CI	OR	95% CI
RHR	< 60 bpm	1.00		1.00	
	60–69	1.09	0.93–1.27	1.12	0.96–1.30
	70–79	1.00	0.85–1.18	1.25	1.06–1.47
	80+	1.28	1.05–1.55	1.18	0.97–1.44
	Linear *P*	0.057		0.116	
	Nonlinear *P*	0.143		0.185	
SDNN	Q1 (lowest)	1.00		1.00	
	Q2	0.81	0.70–0.94	0.91	0.79–1.06
	Q3	0.93	0.80–1.08	0.89	0.77–1.04
	Q4 (highest)	0.83	0.71–0.97	0.91	0.78–1.06
	Linear *P*	0.148		0.182	
	Nonlinear *P*	< 0.001		0.26	
RMSSD	Q1 (lowest)	1.00		1.00	
	Q2	0.82	0.71–0.96	0.87	0.75–1.00
	Q3	0.96	0.83–1.12	0.84	0.72–0.97
	Q4 (highest)	0.90	0.78–1.04	0.82	0.71–0.95
	Linear *P*	0.46		0.041	
	Nonlinear *P*	0.047		0.29	
pNN50	Q1 (lowest)	1.00		1.00	
	Q2	0.89	0.76–1.03	0.85	0.73–0.99
	Q3	0.85	0.73–0.99	0.85	0.74–0.99
	Q4 (highest)	0.85	0.73–0.99	0.88	0.75–1.02
	Linear *P*	0.018		0.139	
	Nonlinear *P*	0.056		0.103	
LF power	Q1 (lowest)	1.00		1.00	
	Q2	0.82	0.70–0.95	0.91	0.79–1.06
	Q3	0.88	0.76–1.03	0.89	0.77–1.04
	Q4 (highest)	0.83	0.72–0.97	0.87	0.75–1.02
	Linear *P*	0.029		0.091	
	Nonlinear *P*	0.017		0.85	
HF power	Q1 (lowest)	1.00		1.00	
	Q2	0.81	0.70–0.95	0.90	0.77–1.04
	Q3	0.81	0.70–0.94	0.91	0.78–1.06
	Q4 (highest)	0.86	0.74–1.00	0.96	0.82–1.11
	Linear *P*	0.18		0.37	
	Nonlinear *P*	0.007		0.56	

Table footnote is the same as Table 2.

**Figure 1:**
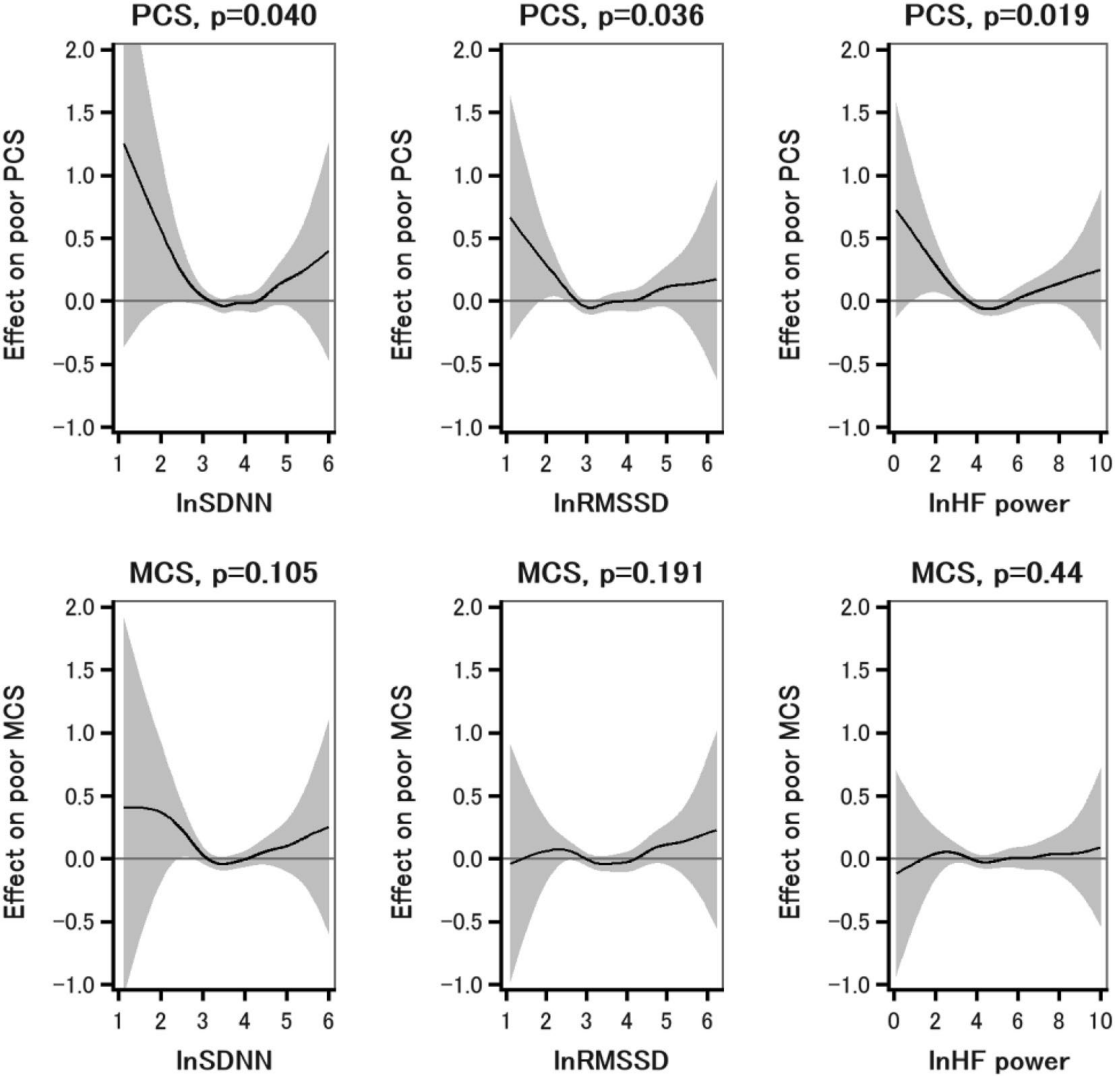
Associations between SDNN, RMSSD, and HF power; and the summary components of SF-8 using the generalized additive model with a smoothing spline [degree of freedom = 3]. Covariates included variables of sex, age, community, BMI, hypertension, diabetes, smoking, regular alcohol drinking, regular exercise, unhealthy sleep duration, and medical history. *PCS* physical component score, *MCS* mental component score, *SDNN* standard deviation of the normal-to-normal intervals, *RMSSD* root mean square of the successive differences of NN intervals, and *HF* high-frequency.

The associations between SDNN, RMSSD, and HF power; and the summary components of SF-8 were illustrated using GAM for nonlinear trends (Fig. 1). The spline lines for poor PCS increased in those who had lower SDNN, RMSSD, and HF power conditions and trended slightly upward in the higher levels. The nonlinear trends were all significant (P for nonlinear trend: 0.040 for SDNN, 0.036 for RMSSD, and 0.019 for HF power). On the contrary, the effect on poor MCS was almost even across the levels of PRV-derived parameters.

## Discussion

We found that PRV-derived parameters, such as SDNN, RMSSD, and HF power were associated with poor HRQOL on the GH, PF, RP, VT, and SF subscales of the SF-8. Factors related to the PCS were more strongly associated compared with those related to the MCS. The association was robust after adjustment for several confounders. RHR was positively correlated with a decrease in HRQOL, mainly represented by GH, PF, and VT. GAM suggested that the relationship between PRV-derived parameters and poor PCS was nonlinear.

Following the interpretation of HRV parameters, SDNN represents overall autonomic function, and RMSSD and HF power reflect parasympathetic regulation, but caution should be used in the interpretation of PRV-derived parameters. We measured resting PRV in participants from the general population. The accuracy of PRV measurements has been reviewed^18^, and PRV was found to be sufficiently accurate as a measure of HRV when measured at rest in healthy subjects. A five-minute measurement of PRV (LF power, HF power, LF/HF ratio) in the present study was moderately correlated with the measurement given from 24-h recordings of HRV^30^. Nevertheless, it is possible that PRV represents the different cardiovascular dynamics than HRV, and further research is needed, including research in relation to autonomic function^20^. Whereas the SF-8 could evaluate HRQOL related to both physical and mental health. HRQOL often decreases due to physical or mental stresses^5,32^ and socioeconomic status^33^.

Although we analyzed our data with a multivariable-adjusted model, including BMI, hypertension, diabetes, smoking, regular alcohol drinking, regular exercise, unhealthy sleep duration, and medical history, these factors did not attenuate the association. This implied that the association was not explained only by the confounders which strongly affect both PRV-derived parameters and HRQOL.

The SF-8 Japanese version was developed as a short version of the SF-36^2^ and the two versions are comparable^34^. Although the two summary scores are classified into the physical and mental aspects, GH and VT were interpreted to be involved with both physical and mental conditions^2^. SF, MH, and RE, which mainly comprise the MCS, also showed a trend toward lower ORs with some higher PRV-derived parameters. Thus, we could not deny the association of poor HRQOL with psychological causes. Vital exhaustion, a psychological stressor, has been associated with decreased SDNN and LF power^35^. PRV-derived parameters might be influenced by psychological factors, probably leading to a decline in self-rated health as represented in the GH or VT subscales.

Of note, for some PRV-derived parameters, higher values were not preferable for HRQOL for reasons that are not well understood. Higher HRV was associated with abnormal heart rate patterns in the elderly which increased the mortality risk^36^. In the present study, individuals with atrial fibrillation on ECG were excluded; thus, this effect was not considered. When values of PRV-derived parameters are very high, physicians should detect some of the causes for lower self-rated health.

Given the well-established link between socioeconomic inequalities and health, the association between PRV and HRQOL may be confounded or mediated by the socioeconomic status or underlying chronic diseases, which we could not access. Socioeconomic status affected psychological stress assessed by depressed HRV conditions^37^, though it will also be required to examine the association using PRV-derived parameters.

Although we analyzed a large population to determine an association between PRV and HRQOL in Japan, several limitations should be mentioned. First, we did not have information on socioeconomic status, such as work, income, and education. Indeed, socioeconomic status affects HRV parameters as a stressor and affects HRQOL^33^. Second, we could not identify the various kinds of illnesses the participants may have had or the medications they were taking, and that possibly influenced PRV. Third, a causal relationship between PRV-derived parameters and HRQOL scores could not be determined, because a possible bi-directional impact was considered in this cross-sectional study. A longitudinal study is needed to investigate this relationship. Finally, a variety of reasons may explain the difference from HRV, including the effect of pulse wave noise, the measurement position, and the location of the measurement sensor^20^. In particular, the pre-processing techniques, including the method of removing noise in the pulse wave signal, are dependent on the measurement device, and the lack of standardization is a major issue.

In conclusion, reduced PRV-derived SDNN, RMSSD and HF power were associated with poor HRQOL in the domain of physical function. Higher levels of these parameters did not necessarily translate into better HRQOL. Data suggest that maintaining PRV-derived parameters in the range indicated as the second or third quartile of PRV-derived parameters is important for good HRQOL. PRV measurement is a useful and non-invasive tool to assess an individual’s perceived health.

### Supplementary Information

Supplementary Table S1.

Supplementary Table S2.

## Data Availability

All data generated or analyzed during this study are included in this published article (and its Supplementary Information files).

## References

[1] Tokuda Y (2009). Assessing items on the SF-8 Japanese version for health-related quality of life: A psychometric analysis based on the nominal categories model of item response theory. Value Health.

[2] Fukuhara S, Ware JE, Kosinski M, Wada S, Gandek B (1998). Psychometric and clinical tests of validity of the Japanese SF-36 health survey. J. Clin. Epidemiol..

[3] Dugan SA (2009). The impact of physical activity level on SF-36 role-physical and bodily pain indices in midlife women. J. Phys. Act. Health.

[4] Sasai T (2010). Effects of insomnia and sleep medication on health-related quality of life. Sleep Med..

[5] Saito I (2006). Impact of diabetes on health-related quality of life in a population study in Japan. Diabet. Res. Clin. Pract..

[6] Kitaoka M (2016). The relationship between hypertension and health-related quality of life: Adjusted by chronic pain, chronic diseases, and life habits in the general middle-aged population in Japan. Environ. Health Prev. Med..

[7] Parra DC (2010). Perceived and objective neighborhood environment attributes and health related quality of life among the elderly in Bogota, Colombia. Soc. Sci. Med..

[8] Tsai SY, Chi LY, Lee CH, Chou P (2007). Health-related quality of life as a predictor of mortality among community-dwelling older persons. Eur. J. Epidemiol..

[9] Nilsson E, Festin K, Lowen M, Kristenson M (2020). SF-36 predicts 13-year CHD incidence in a middle-aged Swedish general population. Qual. Life Res..

[10] Friedman EM, Teas E (2023). Self-rated health and mortality: Moderation by purpose in life. Int. J. Environ. Res. Public Health.

[11] Goldberger JJ, Arora R, Buckley U, Shivkumar K (2019). Autonomic nervous system dysfunction: JACC focus seminar. J. Am. Coll. Cardiol..

[12] Jarczok MN (2022). Heart rate variability in the prediction of mortality: A systematic review and meta-analysis of healthy and patient populations. Neurosci. Biobehav. Rev..

[13] Carnethon MR, Golden SH, Folsom AR, Haskell W, Liao D (2003). Prospective investigation of autonomic nervous system function and the development of type 2 diabetes: The atherosclerosis risk in communities study, 1987–1998. Circulation.

[14] Saito I (2022). Role of insulin resistance in the association between resting heart rate and type 2 diabetes: A prospective study. J. Diabet. Compl..

[15] Wulsin LR, Horn PS, Perry JL, Massaro JM, D'Agostino RB (2015). Autonomic imbalance as a predictor of metabolic risks, cardiovascular disease, diabetes, and mortality. J. Clin. Endocrinol. Metab..

[16] Weinstein G, Davis-Plourde K, Beiser AS, Seshadri S (2021). Autonomic imbalance and risk of dementia and stroke: The framingham study. Stroke.

[17] Chandola T, Heraclides A, Kumari M (2010). Psychophysiological biomarkers of workplace stressors. Neurosci. Biobehav. Rev..

[18] Schafer A, Vagedes J (2013). How accurate is pulse rate variability as an estimate of heart rate variability? A review on studies comparing photoplethysmographic technology with an electrocardiogram. Int. J. Cardiol..

[19] Yuda E (2020). Pulse rate variability: A new biomarker, not a surrogate for heart rate variability. J. Physiol. Anthropol..

[20] Mejia-Mejia E, May JM, Torres R, Kyriacou PA (2020). Pulse rate variability in cardiovascular health: A review on its applications and relationship with heart rate variability. Physiol. Meas..

[21] Jylha M (2009). What is self-rated health and why does it predict mortality? Towards a unified conceptual model. Soc. Sci. Med..

[22] Kanbara K, Morita Y, Hasuo H, Abe T (2021). The association between heart rate variability and quality of life in patients with functional somatic syndrome and healthy controls. Appl. Psychophysiol. Biofeedback.

[23] Meule A (2013). Quality of life, emotion regulation, and heart rate variability in individuals with intellectual disabilities and concomitant impaired vision. Psychol. Well-Being: Theory Res. Pract..

[24] Kim D (2023). Association between health-related quality of life and heart rate variability in elderly individuals with cognitive impairment in Korea: Cross-sectional study. BMC Geriatr..

[25] Lu WC (2016). Correlation between health-related quality of life in the physical domain and heart rate variability in asymptomatic adults. Health Qual Life Outcomes.

[26] Andreasson A, Axelsson J, Bosch JA, Balter LJ (2021). Poor sleep quality is associated with worse self-rated health in long sleep duration but not short sleep duration. Sleep Med..

[27] Kim JK, Ahn JM (2019). Digital IIR filters for heart rate variability: A comparison between butterworth and elliptic filters. Eng. Med. Comput. Sci..

[28] Task Force of the European Society of Cardiology and the North American Society of Pacing and Electrophysiology (1996). Heart rate variability: Standards of measurement, physiological interpretation, and clinical use. Eur. Heart J..

[29] Ahn JM (2013). Heart rate variability (HRV) analysis using simultaneous handgrip electrocardiogram and fingertip photoplethysmogram. Adv. Inf. Sci. Serv. Sci. (AISS).

[30] Saito I (2018). Association Between heart rate variability and home blood pressure: The toon health study. Am. J. Hypertens..

[31] Benedetti A, Abrahamowicz M (2004). Using generalized additive models to reduce residual confounding. Stat. Med..

[32] Du R (2020). Health-related quality of life and associated factors in patients with myocardial infarction after returning to work: A cross-sectional study. Health Qual Life Outcomes.

[33] Hemingway H (2005). Does autonomic function link social position to coronary risk? The Whitehall II study. Circulation.

[34] Fukuhara S, Suzukamo Y (2005). Instruments for measuring health-related quality of life: SF-8 and SF-36. Igakuno ayumi.

[35] Shah AS (2021). Association of psychosocial factors with short-term resting heart rate variability: The atherosclerosis risk in communities study. J. Am. Heart Assoc..

[36] Stein PK, Domitrovich PP, Hui N, Rautaharju P, Gottdiener J (2005). Sometimes higher heart rate variability is not better heart rate variability: Results of graphical and nonlinear analyses. J. Cardiovasc Electrophysiol..

[37] Lampert R, Ickovics J, Horwitz R, Lee F (2005). Depressed autonomic nervous system function in African Americans and individuals of lower social class: A potential mechanism of race- and class-related disparities in health outcomes. Am. Heart J..

