# Supplementary material for: Pulse rate variability and health-related quality of life assessment with the Short Form-8 Japanese version in the general Japanese population
Source: Sci Rep. 2024 Feb 20;14:4157. doi: 10.1038/s41598-024-54748-9 (PMC10879517; doi:10.1038/s41598-024-54748-9)
Supplement: Supplementary file 1 — Supplementary Table S1. [file 41598_2024_54748_MOESM1_ESM.docx]

S1 Table. Sex- and age-adjusted ORs and 95% CIs according to RHR levels and quartile of PRV parameters for poor general health, physical function, role physical, and bodily pain (n=5,908)

|  |  | Subscale of SF-8 (Sex and age-adjusted models) | | | | | | | |
| --- | --- | --- | --- | --- | --- | --- | --- | --- | --- |
|  |  | General health | | Physical function | | Role physical | | Bodily pain | |
| Parameter | Category | OR | 95% CI | OR | 95% CI | OR | 95% CI | OR | 95% CI |
| RHR | <60 bpm | 1.00 |  | 1.00 |  | 1.00 |  | 1.00 |  |
|  | 60-69 | 1.12 | 0.93-1.34 | 1.14 | 0.98-1.33 | 1.06 | 0.91-1.23 | 1.01 | 0.87-1.18 |
|  | 70-79 | 1.20 | 0.99-1.46 | 1.08 | 0.92-1.27 | 1.08 | 0.92-1.27 | 0.99 | 0.84-1.16 |
|  | 80+ | 1.26 | 1.01-1.59 | 1.25 | 1.03-1.52 | 1.19 | 0.98-1.44 | 1.03 | 0.85-1.24 |
|  | Linear *P* | 0.010 | | 0.023 | | 0.023 | | 0.88 | |
|  | Non-linear *P* | 0.43 | | 0.07 | | 0.141 | | 0.91 | |
| SDNN | Q1 (lowest) | 1.00 |  | 1.00 |  | 1.00 |  | 1.00 |  |
|  | Q2 | 0.82 | 0.69-0.97 | 0.80 | 0.69-0.93 | 0.79 | 0.68-0.91 | 0.92 | 0.79-1.06 |
|  | Q3 | 0.82 | 0.69-0.98 | 0.89 | 0.77-1.03 | 0.91 | 0.79-1.06 | 0.94 | 0.81-1.08 |
|  | Q4 (highest) | 0.87 | 0.73-1.03 | 0.82 | 0.71-0.96 | 0.77 | 0.66-0.90 | 0.95 | 0.82-1.11 |
|  | Linear *P* | 0.92 | | 0.32 | | 0.051 | | 0.81 | |
|  | Non-linear *P* | <0.001 | | <0.001 | | <0.001 | | 0.78 | |
| RMSSD | Q1 (lowest) | 1.00 |  | 1.00 |  | 1.00 |  | 1.00 |  |
|  | Q2 | 0.87 | 0.73-1.03 | 0.81 | 0.70-0.93 | 0.88 | 0.76-1.02 | 0.92 | 0.80-1.06 |
|  | Q3 | 0.89 | 0.75-1.06 | 0.83 | 0.72-0.96 | 0.89 | 0.77-1.03 | 1.01 | 0.87-1.17 |
|  | Q4 (highest) | 0.84 | 0.71-1.00 | 0.91 | 0.79-1.05 | 0.91 | 0.79-1.05 | 0.96 | 0.83-1.10 |
|  | Linear *P* | 0.63 | | 0.95 | | 0.34 | | 0.60 | |
|  | Non-linear *P* | 0.001 | | <0.001 | | <0.001 | | 0.55 | |
| pNN50 | Q1 (lowest) | 1.00 |  | 1.00 |  | 1.00 |  | 1.00 |  |
|  | Q2 | 0.96 | 0.81-1.14 | 0.83 | 0.72-0.96 | 0.84 | 0.73-0.98 | 1.09 | 0.94-1.26 |
|  | Q3 | 0.94 | 0.79-1.11 | 0.87 | 0.75-1.01 | 0.87 | 0.75-1.00 | 0.98 | 0.85-1.14 |
|  | Q4 (highest) | 0.77 | 0.65-0.92 | 0.82 | 0.71-0.95 | 0.85 | 0.73-0.99 | 1.01 | 0.87-1.17 |
|  | Linear *P* | 0.002 | | 0.003 | | 0.010 | | 0.53 | |
|  | Non-linear *P* | 0.34 | | 0.009 | | 0.002 | | 0.58 | |
| LF power | Q1 (lowest) | 1.00 |  | 1.00 |  | 1.00 |  | 1.00 |  |
|  | Q2 | 0.75 | 0.63-0.89 | 0.75 | 0.65-0.87 | 0.84 | 0.73-0.97 | 0.91 | 0.78-1.05 |
|  | Q3 | 0.76 | 0.63-0.90 | 0.80 | 0.69-0.93 | 0.85 | 0.73-0.99 | 0.84 | 0.73-0.98 |
|  | Q4 (highest) | 0.79 | 0.66-0.94 | 0.81 | 0.70-0.94 | 0.81 | 0.69-0.94 | 0.90 | 0.77-1.04 |
|  | Linear *P* | 0.121 | | 0.064 | | 0.022 | | 0.116 | |
|  | Non-linear *P* | <0.001 | | <0.001 | | 0.009 | | 0.40 | |
| HF power | Q1 (lowest) | 1.00 |  | 1.00 |  | 1.00 |  | 1.00 |  |
|  | Q2 | 0.75 | 0.63-0.89 | 0.74 | 0.64-0.86 | 0.77 | 0.67-0.89 | 0.84 | 0.73-0.98 |
|  | Q3 | 0.77 | 0.65-0.92 | 0.81 | 0.70-0.94 | 0.79 | 0.68-0.92 | 0.83 | 0.71-0.96 |
|  | Q4 (highest) | 0.82 | 0.69-0.98 | 0.87 | 0.75-1.01 | 0.85 | 0.74-0.99 | 0.88 | 0.76-1.02 |
|  | Linear *P* | 0.52 | | 0.72 | | 0.181 | | 0.187 | |
|  | Non-linear *P* | <0.001 | | <0.001 | | <0.001 | | 0.51 | |

ORs were adjusted for sex and age stratified by community. Ranges: SDNN (ms) Q1, <3.30 (log), Q2, 3.30-3.58, Q3, 3.59-3.91, Q4, 3.92+; RMSSD (ms) Q1, <3.00 (log), Q2, 3.00-3.32, Q3, 3.33-3.73, Q4. 3.74+; pNN50 (%) Q1, <37, Q2, 37-50, Q3, 51-62, Q4, 63+; LF (ms^2^) Q1, <4.23 (log), Q2, 4.23-4.94, Q3, 4.95-5.80, Q4, 5.81+; and HF (ms^2^) Q1, <3.83 (log), Q2, 3.83-4.50, Q3: 4.51-5.30, Q4, 5.31+. Abbreviations: OR, odds ratio; CI, confidence interval; RHR, resting heart rate; PRV, pulse rate variability; SDNN, standard deviation of the normal-to-normal intervals; RMSSD, root mean square of the successive differences of NN intervals; pNN50, percentage differences between normal NN intervals >50 ms; LF, low-frequency; and HF, high-frequency.
