# Supplementary material for: Pulse rate variability and health-related quality of life assessment with the Short Form-8 Japanese version in the general Japanese population
Source: Sci Rep. 2024 Feb 20;14:4157. doi: 10.1038/s41598-024-54748-9 (PMC10879517; doi:10.1038/s41598-024-54748-9)
Supplement: Supplementary file 2 — Supplementary Table S2. [file 41598_2024_54748_MOESM2_ESM.docx]

S2 Table. Sex- and age-adjusted ORs and 95% CIs according to RHR levels and quartile of PRV parameters for poor validity, social functioning, mental health, and role emotional (n=5,908)

|  |  | Subscale of SF-8 (Sex and age-adjusted models) | | | | | | | |
| --- | --- | --- | --- | --- | --- | --- | --- | --- | --- |
|  |  | Vitality | | Social functioning | | Mental health | | Role emotional | |
| Parameter | Category | OR | 95% CI | OR | 95% CI | OR | 95% CI | OR | 95% CI |
| RHR | <60 bpm | 1.00 |  | 1.00 |  | 1.00 |  | 1.00 |  |
|  | 60-69 | 1.16 | 1.00-1.35 | 1.02 | 0.87-1.19 | 1.16 | 1.00-1.35 | 1.02 | 0.87-1.19 |
|  | 70-79 | 1.32 | 1.12-1.55 | 1.11 | 0.94-1.32 | 1.32 | 1.12-1.55 | 1.11 | 0.94-1.32 |
|  | 80+ | 1.56 | 1.29-1.89 | 1.09 | 0.90-1.34 | 1.56 | 1.29-1.89 | 1.09 | 0.90-1.34 |
|  | Linear *P* | <0.001 | | 0.133 | | 0.071 | | 0.123 | |
|  | Non-linear *P* | 0.97 | | 0.59 | | 0.45 | | 0.121 | |
| SDNN | Q1 (lowest) | 1.00 |  | 1.00 |  | 1.00 |  | 1.00 |  |
|  | Q2 | 0.83 | 0.71-0.96 | 0.89 | 0.76-1.03 | 0.83 | 0.71-0.96 | 0.89 | 0.76-1.03 |
|  | Q3 | 0.84 | 0.72-0.97 | 0.96 | 0.82-1.12 | 0.84 | 0.72-0.97 | 0.96 | 0.82-1.12 |
|  | Q4 (highest) | 0.85 | 0.73-0.98 | 0.94 | 0.81-1.10 | 0.85 | 0.73-0.98 | 0.94 | 0.81-1.10 |
|  | Linear *P* | 0.092 | | 0.48 | | 0.138 | | 0.140 | |
|  | Non-linear *P* | <0.001 | | <0.001 | | 0.053 | | <0.001 | |
| RMSSD | Q1 (lowest) | 1.00 |  | 1.00 |  | 1.00 |  | 1.00 |  |
|  | Q2 | 0.83 | 0.71-0.96 | 0.92 | 0.79-1.07 | 0.83 | 0.71-0.96 | 0.92 | 0.79-1.07 |
|  | Q3 | 0.89 | 0.77-1.03 | 0.89 | 0.76-1.04 | 0.89 | 0.77-1.03 | 0.89 | 0.76-1.04 |
|  | Q4 (highest) | 0.82 | 0.71-0.95 | 0.94 | 0.81-1.09 | 0.82 | 0.71-0.95 | 0.94 | 0.81-1.09 |
|  | Linear *P* | 0.116 | | 0.55 | | 0.069 | | 0.054 | |
|  | Non-linear *P* | 0.002 | | 0.002 | | 0.124 | | 0.091 | |
| pNN50 | Q1 (lowest) | 1.00 |  | 1.00 |  | 1.00 |  | 1.00 |  |
|  | Q2 | 0.92 | 0.79-1.07 | 0.85 | 0.73-0.99 | 0.92 | 0.79-1.07 | 0.85 | 0.73-0.99 |
|  | Q3 | 0.86 | 0.75-1.00 | 0.89 | 0.76-1.03 | 0.86 | 0.75-1.00 | 0.89 | 0.76-1.03 |
|  | Q4 (highest) | 0.81 | 0.70-0.94 | 0.85 | 0.73-0.99 | 0.81 | 0.70-0.94 | 0.85 | 0.73-0.99 |
|  | Linear *P* | 0.001 | | 0.033 | | 0.084 | | 0.064 | |
|  | Non-linear *P* | 0.35 | | 0.043 | | 0.52 | | 0.67 | |
| LF power | Q1 (lowest) | 1.00 |  | 1.00 |  | 1.00 |  | 1.00 |  |
|  | Q2 | 0.85 | 0.74-0.99 | 0.86 | 0.74-1.00 | 0.85 | 0.74-0.99 | 0.86 | 0.74-1.00 |
|  | Q3 | 0.87 | 0.75-1.01 | 0.86 | 0.74-1.01 | 0.87 | 0.75-1.01 | 0.86 | 0.74-1.01 |
|  | Q4 (highest) | 0.87 | 0.75-1.01 | 0.80 | 0.68-0.93 | 0.87 | 0.75-1.01 | 0.80 | 0.68-0.93 |
|  | Linear *P* | 0.042 | | 0.030 | | 0.030 | | 0.041 | |
|  | Non-linear *P* | <0.001 | | 0.041 | | 0.24 | | 0.175 | |
| HF power | Q1 (lowest) | 1.00 |  | 1.00 |  | 1.00 |  | 1.00 |  |
|  | Q2 | 0.75 | 0.65-0.87 | 0.87 | 0.75-1.02 | 0.75 | 0.65-0.87 | 0.87 | 0.75-1.02 |
|  | Q3 | 0.82 | 0.71-0.95 | 0.91 | 0.78-1.06 | 0.82 | 0.71-0.95 | 0.91 | 0.78-1.06 |
|  | Q4 (highest) | 0.81 | 0.70-0.94 | 0.94 | 0.80-1.09 | 0.81 | 0.70-0.94 | 0.94 | 0.80-1.09 |
|  | Linear *P* | 0.058 | | 0.48 | | 0.197 | | 0.21 | |
|  | Non-linear *P* | <0.001 | | 0.026 | | 0.28 | | 0.23 | |

S2 Table footnote is the same as S1 Table.
